# Data integration and exploration for the identification of molecular mechanisms in tumor-immune cells interaction

**DOI:** 10.1186/1471-2164-11-S1-S7

**Published:** 2010-02-10

**Authors:** Bernhard Mlecnik, Fatima Sanchez-Cabo, Pornpimol Charoentong, Gabriela Bindea, Franck Pagès, Anne Berger, Jerome Galon, Zlatko Trajanoski

**Affiliations:** 1 Institute for Genomics and Bioinformatics, Graz University of Technology, Petersgasse 14, 8010 Graz, Austria; 2 INSERM, U872, Integrative Cancer Immunology, Paris, France; 3 Genomics Unit, Spanish National Centre for Cardiovascular Research, Madrid, Spain; 4 AP-HP, Georges Pompidou European Hospital, Paris, France

## Abstract

Cancer progression is a complex process involving host-tumor interactions by multiple molecular and cellular factors of the tumor microenvironment. Tumor cells that challenge immune activity may be vulnerable to immune destruction. To address this question we have directed major efforts towards data integration and developed and installed a database for cancer immunology with more than 1700 patients and associated clinical data and biomolecular data. Mining of the database revealed novel insights into the molecular mechanisms of tumor-immune cell interaction. In this paper we present the computational tools used to analyze integrated clinical and biomolecular data. Specifically, we describe a database for heterogenous data types, the interfacing bioinformatics and statistical tools including clustering methods, survival analysis, as well as visualization methods. Additionally, we discuss generic issues relevant to the integration of clinical and biomolecular data, as well as recent developments in integrative data analyses including biomolecular network reconstruction and mathematical modeling.

## Background

Despite extensive characterization of environmental and intrinsic and underlying mechanisms [1,2], markers of the oncogenic process remain so far poorly predictive of patient survival and fail to prove their reliability in clinical use. For example, colorectal cancer is one of the most common malignancies for both men and women [3]. The rate of localized cancers (stage I-II; UICC-TNM classification) is about 40% [4,5]. Despite surgery with curative intent, the risk of recurrence of these early-stage patients is high (approximately 20-30%). To subject all of these patients to post-operative chemotherapy may be inappropriate and costly [6]. Genetic and molecular tumor prognostic factors have been proposed to identify patients who may be at risk for recurrence. None has yet been sufficiently informative for inclusion in clinical practice [5]. Identification of patients with high-risk of recurrence is therefore a major clinical issue. However, in order to develop stratified or personalized strategies for such complex multifactorial disease it is of importance to understand how numerous and diverse elements function together in human pathology. A comprehensive understanding of cancer requires the integration and analysis of data not only from the tumor but also its microenvironment including the immune cells.

Tumors are composed of a complex network of tumor cells, immune cells, stromal components including fibroblasts, and a complex vasculature. To grow, invade, and metastasize, a tumor interacts with its microenvironment, composed of diverse cells of various origins. The microenvironment contains cells of the immune system, including inflammatory infiltrates of innate immunity and infiltrates of the adaptive immune response. In colorectal cancer, previous studies have suggested a clinical role of the immune infiltrates [7-11]. In order to investigate the role of the immune infiltrates and analyze the tumor immunological microenvironment in humans we developed and installed a database for cancer immunology with more than 1700 patients and associated clinical data and biomolecular data. By analyzing the data we showed the importance of early-metastatic invasion in colorectal cancer and could pinpoint a novel prognostic marker for survival [10]. We evidenced that the recently characterized immune cell subpopulation of effector-memory T cells (T_EM_), may have a central role in the control of tumor spreading to lymphovascular and perineural structures but also to lymph node or distant organs. In subsequent study we demonstrated the role of the adaptive immune system for predicting clinical outcome [9]. Furthermore, we revealed the importance for patient prognosis of the nature, the functional orientation, the density and the localization of immune cell populations within the primary tumor. Thus, adaptive immune reaction and intratumoral T-cell subpopulations were better predictor of survival than traditional staging based on a cancer's size and spread [9].

In the light of these studies it was of utmost importance to integrate the data and develop tools for analysis and visualization. In this paper, we present the solutions developed to analyze the tumor immunological microenvironment in humans including database, analytical tools, and tools for visualization. Specifically, we describe here the database for clinical and biomolecular data, the interfacing bioinformatics and statistical tools including clustering methods, survival analysis, as well as visualization methods. Furthermore, we discuss upcoming developments for integrative data analyses including biomolecular network reconstruction and mathematical modeling.

## Bioinformatics and statistics tools for cancer immunology

### Database for cancer immunology

The database developed for cancer immunology (Tumor Microenvironment (TME)) integrates clinical and biomolecular data. The underlying relational database model is designed as a cancer patient oriented database which takes all the patients anamnesis and clinical and medical history information into account whereby all patients are linked to a speci?c hospital. Security issues were treated in regard to the interest of patients. Ethical, Legal and Social Implications (ELSI) have been fulfilled (agreement #903434), security modules implemented, and anonymous information stored. The patient information additionally includes medical problems, surgery and detailed cancer information. Additionally TME.db allows the storage of a variety of different high-throughput experiments including:

• Real-Time TaqMan qPCR gene expression data (Low density arrays, single probes, T-cell repertoire analysis)

• Microsatellite instability (MSI) and mutations data

• Flow cytometric (FACS) phenotyping data

• Protein quantification (ELISA, Quantibody, cytometric beads assays) data

• Functional data (proliferation, survival, apoptosis, migration assays)

• Immunohistochemical data (Tissue Micro Array (TMA) and whole slide analysis)

TME.db joins and integrates all different types of data and stores them in a common place where all the determined analysis parameters are linked in a clear way dependent on the sample material and the experiment type. For accessing all the stored information again sophisticated query methods were developed in order to retrieve the data in a pre-modi?ed way, already prepared for statistical analysis. As of May 2009, the database incorporates 1784 patients with associated clinical data with 60 parameters (e.g. tumor staging, treatment, cancer relapse) and 16400 different material information as well as biomolecular measurements (including qPCR for 400 genes from 125 patients, 820 FACS parameters from 40 patients, 20 tissue microarray assays for 600 patients).

#### Software architecture

TME is a multi-tier client-server application and can be subdivided into different functional modules which interact as self-contained units according to their defined responsibilities: presentation tier, business tier and runtime environment. The presentation tier within TME is formed by a Web interface, which allows programming access to parts of the application logic. Thus, on the client side, a user requires an Internet connection and a recent Web browser with Java support, available for almost every platform. The business tier is realized as view-independent application logic, which stores and retrieves datasets by communicating with the persistence layer. The internal management of files is also handled from a central service component, which persists the meta-information for acquired files to the database. All services of this layer are implemented as STRUTS and are using SITEMESH.

#### Model driven development

In order to reduce coding and to increase the long term maintainability, the model driven development environment AndroMDA is used to generate components of the persistence layer and recurrent parts from the above mentioned business layer. AndroMDA accomplishes this by translating an annotated UML-model into a JEE-platform-specific implementation using Enterprise Java Beans (EJB), STRUTS and SITEMESH. Due to the flexibility of AndroMDA, application external services, such as the user management system, have a clean integration in the model. Dependencies of internal service components on such externally defined services are cleanly managed by its build system. By changing the build parameters in the AndroMDA configuration, it is also possible to support different relational database management systems. This is because platform specific code with the same functionality is generated for data retrieval. Furthermore, technology lock-in regarding the implementation of the service layers was also addressed by using AndroMDA, as the implementation of the service facade can be switched during the build process from Spring based components to distributed Enterprise Java Beans. At present, TME is operating on one local machine and, providing the usage scenarios do not demand it, this architectural configuration will remain. However, chosen technologies are known to work on Web server farms and crucial distribution of the application among server nodes is transparently performed by the chosen technologies.

#### Data retrieval, collaboration and data sharing

TME offers search masks which allow keyword based searching in the recorded projects, experiments and notes. These results are often discussed with collaboration partners to gain different opinions on the same raw data. In order to allow direct collaboration between scientists TME is embedded into a central user management system which offers multiple levels of access control to projects and their associated experimental data. The sharing of projects can be done on a per-user basis or on an institutional basis. For small or local single-user installations, the fully featured user management system can be replaced by a file-based user management which still offers the same functionalities from the sharing point of view, but lacks institute-wide functionalities.

### Bioinformatics analysis tools

The database was mined using standard bioinformatics tools. Specifically, qPCR and FACS data were explored using two-dimensional hierarchical clustering of correlation matrices (i.e. gene-wise correlation of the respective patient groups [9]). Genesis clustering software was used to visualize the correlation matrix and to perform Pearson un-centered hierarchical clustering [12]. This tool was developed for large-scale gene expression cluster analysis and integrates various tools for microarray data analysis such as filters, normalization and visualization tools, distance measures as well as common clustering algorithms including hierarchical clustering, self-organizing maps, k-means, principal component analysis, and support vector machines [12].

### Statistical analysis

Survival analysis provides a statistical framework for the modeling and statistical analysis of the time to event for a cohort of patients [13]. Since the distribution of survival times might have an unusual and often unknown form, nonparametric Kaplan-Meier estimates are widely used when censoring is present for the characterization of groups of patients with different underlying characteristics, i.e. calculating median survival times and patients at risk after a given period. Similarly, the log-rank non-parametric test is used to check the null hypothesis that at any time point there is no difference in the probability of the event of interest between the groups [14]. The magnitude of the difference and its confidence interval can be calculated using a Cox proportional hazards model. Furthermore the effect of a novel biomarker can be adjusted for traditional parameters if this modeling strategy is used on several covariates.

TME implements the previous tests within a statistical analysis module. Calculations are done using the survival package from R [15] to which TME connects using RServe [16]. The aim is the automatic detection of biomarkers or sets of biomarkers that - alone or in combination with other parameters - are able to discriminate groups of colorectal cancer patients with good prognosis from those with bad prognosis for both, overall and disease-free survival. In particular, TME provides:

- Kaplan-Meier curves, estimates of the median survival time and number of patients at risk after a certain time period for the different groups of patients

- Log-rank test for the analysis of the differences in survival between groups of patients with different underlying characteristics

- Univariate Cox proportional hazards model to estimate the magnitude of the effect of the covariate in survival

- Tools for the categorization of numeric covariates into a fixed number of levels. This can be useful for the classification of the patients into groups based on the biomolecular markers stored in TME for each patient, such as the expression level of a gene or the number of cells of a given type found at different locations of the tumor sample.

Although categorization of the patients into groups might result in loss of information [17], this is often done in clinical practice. The way the cut-off is set for dichotomizing a continuous variable is also controversial: A previously described value or a biologically justified level can be used as suggested by Altman *et al *[18]. In the absence of a biologically sound cut-off value, using a statistic of the sample (such as the median) balances the number of cases per group but results in different levels across studies making the comparison of results from different groups difficult [17]. Hence, the analysis must be repeated in an independent cohort of patients categorized using the cut-off previously selected. The same is true when using the "minimum p-value" approach [19], i.e. taking the point yielding the "maximum" significance between groups. This approach has additional important problems such as the overestimation of the prognostic importance of the covariate and multiple testing issues that might be accounted for [18]

TME allows the inspection of the covariates dichotomizing them based in any of the previous options. In particular, if the minimum p-value approach is used the log-rank p-value can be corrected using either the formula proposed by Altman *et al *[18] or with cross-validation as proposed by Faraggi & Simon [20]. Additionally, TME implements the shrinkage method proposed by Holländer *et al *[21] to correct the hazard ratios.

Next version of TME will also include multivariate analysis using a Cox proportional hazards model and decision trees, which can easily accommodate heterogeneous variables and have yielded already satisfactory results in the discovery of biomarkers for breast cancer [22].

### Data visualization

Data visualization was carried out using the publicly available software tools Cytoscape, ClueGO, and GOlorize. Cytoscape is free software package for visualizing, modeling and analyzing molecular and genetic interaction networks [23-26]. In Cytoscape, the nodes represent genes or proteins and they are connected with edges which representing interactions. Typical biological networks at the molecular level are gene regulation networks, signal transduction networks, protein interaction networks, and metabolic networks. In order to capture biological information, ClueGO [25], a Cytoscape plug-in, uses Gene Ontology [27] categories that are overrepresented in selected one or two lists of genes. ClueGO takes advantage of GOlorize [26] plug-in, an efficient tool to the same class node-coloring and the class-directed layout algorithm for advanced network visualization.

## Discussion

In this paper we described computational tools developed specifically to address biological questions in cancer immunology. The computational tools include: 1) a database for clinical and biomolecular data comprising >1700 patients with associated clinical information, FACS data, qPCR data, tissue microarray data; 2) bioinformatics tools developed for the analyses of medium and large-scale data, 3) statistical tools for the survival analysis; and 4) tools for visualization of the data. The power of the dedicated informatics solution is leveraged by the integration of all computational resources using various interfaces. During the course of the development of the database, the implementation of the analytical tools, and the analysis of the data we have learned several important lessons.

### Lessons learned

First, development of a dedicated database is time-consuming but indispensable task. In recent years, the biology community has expended considerable effort to confront the challenges of managing heterogeneous data in a structured and organized way and as a result developed information management systems for both raw and processed data. Laboratory information management systems (LIMS) have been implemented for handling data entry from robotic systems and tracking samples as well as data management systems for processed data including microarrays, proteomics data, and microscopy data. In general, these sophisticated systems are able to manage and analyze data generated for only a single type or a limited number of instruments, and were designed for only a specific type of molecule. Thus, addressing a biological question relying on several complementary technologies requires a specific off-the-shelf database. It should be noted that such a database could absorb several person-years of software engineering and this effort tends to be underestimated.

Second, incorporation of clinical data poses additional challenges. Many institutions have electronic patient records and in principle, extracting the information could be straightforward. However, technical, ethical, and legal issues might delay or even prohibit the process of data collection. Heterogeneous clinical and departmental information systems, accessibility of patient data, and managing sensitive information can introduce several levels of complexity and require extensive stakeholder discussions. A complex information management system that captures in a secure way the relevant data is suggestive only for large (i.e. several hundred PIs) institutions. The majority of the labs are better off with a design of a relatively small, departmental database for only few specific cohorts. The patient data should be first de-identified and then provided to the biologists and bioinformaticians.

Third, primary data should be archived at a separate location and only preprocessed and normalized data should be stored in the dedicated database. Although it is tempting to upload and analyze all types of data in a single system, experience shows that primary data is mostly used once. This approach is even more advisable for large-scale data including microarrays, proteomics of sequence data. However, links to the primary data need to be secured so that later re-analyses using improved tools can be guaranteed. In this context it is noteworthy that in the analyses we have performed so far only medium-throughput data was used, meaning that the number of analyzed molecular species was in the range of 100-1000. With this number of elements the majority of the tools perform satisfactorily on a standard desktop computer. Performance is a crucial issue if the number of molecules detected in a single patient sample increases to >10.000 (like in microarray studies) or >100.000 (proteomics studies) and the used methods need to be re-evaluated.

In this paper we show a powerful approach for integrative analyses of heterogenous biomolecular data and clinical data. Although powerful, our approach was sequential, i.e. the data was integrated in the database and the query masks allowed sequential analyses of specific biomolecular data, and their correlation with clinical data. We strongly believe that integrative data analyses methods will provide additional insights otherwise hidden in the complex data sets. Several approaches were suggested previously (e.g. [23-26,28-30]). However, normalization of the data, availability of reference datasets, and scarcity of the data (specific measurements are not available for all patients) are non-trivial issues which are difficult to address. In this context, novel data integration approaches are highly desirable. In the following paragraphs we highlight two approaches, namely biomolecular network reconstruction and mathematical modelling, which have the potential to provide mechanistic insights and ultimately translation of this knowledge to clinical applications.

### Biomolecular network reconstruction

One emerging field, which was not addressed in this paper is biomolecular network reconstruction. The data we have so far used are actual measurements and are limited to the available technology and/or samples. There is a wealth of information stored in public databases on protein-protein interactions, text mining, two-hybrid screens, or gene silencing using siRNA. The integration of this datasets in databases like STRING [31] and the visualization tools like Cytoscape [23] and associated-software such as ClueGO [25] opens new avenues of exploration of biomolecular networks.

### Mathematical modeling

Since the pathophysiological mechanisms underlying cancer are highly complex and involve many different cell types and processes, mathematical modeling is becoming an important tool to integrate the biological information and enhance our understanding of interaction between cancer and immune system. Moreover, mathematical modeling may direct direction of experimental work for treatment and diagnosis. Here we briefly describe relevant modeling efforts for tumour-immune cells interaction.

#### Mathematical models of cancer

Traditionally, mathematical models of cancer fall into two broad camps: descriptive and mechanistic [32]. Descriptive models tend to focus on reproducing the gross characteristic of tumors such as size and cell numbers, are generally used to investigate tumor cell population dynamics, without emphasis on cell biological detail [32-34]. Over the last decades, many mathematical models have been proposed that focus on tumor growth. Macklin *et al. *[35] performed a new multiscale mathematical model for solid tumor growth which couples an improved model of tumor invasion with a model of tumor-induced angiogenesis. A large number of studies have described deterministic models which have been used to model the spatio-temporal spread of tumors [36]. By contrast, mechanistic models focus on specific aspects of tumor progression in order to explain the underlying biological processes that drive them [32,33,37].

#### Mathematical models of immune response

The regulation of immune system involves the interaction between populations of pathogen and immune cell. Immunological memory and specificity are property of the immune system. This ability to respond more rapidly and effective than to the first exposure [38]. Understanding of these aspects requires quantitative models of proliferation and differentiation of T lymphocytes. Mathematical modeling can describe these behaviors as deterministic or stochastic models. De Boer *et al*. proposed the simple mathematical model in which parameters can be estimated (proliferation and death rate) during clonal expansion and contraction phase [39,40]. Three models have been proposed by Ganusov [41] to discriminate between alternative memory cell differentiation pathways.

#### Mathematical models of cancer-immune interactions

Mathematical modeling of tumor growth that includes the immune response and chemotherapy treatment would provide an analytical predictive framework. Kim *et al*. developed a mathematical model with the new experimental data to gain insights into the dynamics and potential impact of the resulting anti-leukemia immune response on chronic myelogenous leukemia (CML) [42]. Moore *et al*. modeled the interaction T cell subpopulations and CML cancer cells in the body, using a system of ordinary differential equations [43]. Steffen *et al*. presented a mathematical model of melanoma invasion into healthy tissue with an immune response. They used this model as a framework with which to investigate primary tumor invasion and treatment by surgical excision [44].

## Conclusion

In this paper we presented computational tools developed to manage and explore clinical and biomolecular data for the identification of molecular mechanisms in the tumor microenvironment. The presented bioinformatics and statistics solutions were applied on a patient cohort with colorectal cancer and revealed novel insights in the tumor-immune cells interaction. Although used to address a specific question, the approach is generic and can be applied also to different cancers as well as to other multifactorial diseases like diabetes or cardiovascular diseases.

## List of abbreviations used

JavaEE: Java Enterprise Edition platform; MDA: Model Driven Architecture; SOAP: Simple Object Access Protocol

## Competing interests

The authors declare that they have no competing interests.

## Authors' contributions

BM developed the database. BM, FSC, GB, and PC carried out the analyses. FP and AB collected and annotated the clinical data. JG and ZT coordinated the project. All authors contributed to the drafting of the manuscript, and read and approved the final manuscript.
